# Association of low-carbohydrate-diet score and osteoporotic fractures: National Health and Nutrition Examination Survey

**DOI:** 10.3389/fpubh.2025.1668024

**Published:** 2025-10-08

**Authors:** Yuntao Shen, Hebao Wei

**Affiliations:** ^1^Department of Orthopedics, Haining People's Hospital, Haining, Zhejiang, China; ^2^Department of Orthopedics, Jiaxing Xiuzhou District People's Hospital, Jiaxing, Zhejiang, China

**Keywords:** low-carbohydrate diet, osteoporotic fractures, National Health and Nutrition Examination Survey, macronutrients, adults and older adults

## Abstract

**Background:**

Osteoporotic fractures represent a significant public health concern on a global scale. There is currently a lack of research on the association between low-carbohydrate-diet score and Osteoporotic fractures risk.

**Methods:**

A cross-sectional analysis was performed involving 13,025 participants from the National Health and Nutrition Examination Survey, utilizing data collected from the years 2005 to 2010, 2013 to 2014, and 2017 to 2018. Logistic regression analyses were used to explore the association between the Low-Carbohydrate Diet score and Osteoporotic fractures risk. Restricted cubic spline analysis was conducted to evaluate the linearity or nonlinearity of the association. Subgroup and interaction analyses were also performed.

**Results:**

Following the adjustment for confounding variables, a positive correlation was identified between elevated Low-Carbohydrate Diet scores and an increased risk of Osteoporotic fractures. Specifically, a one-point increment in Low-Carbohydrate Diet score corresponded to a 1.13% rise in Osteoporotic fractures risk (OR = 1.0113, 95% CI: 1.0015–1.0212, *p* = 0.0240). The risk of Osteoporotic fractures among individuals in the highest Low-Carbohydrate Diet quartile was significantly greater compared to those in the lowest quartile (OR = 1.2248, 95% CI: 1.0212–1.4388, *p* = 0.0295). The Restricted cubic spline analyses revealed a linear relationship between Low-Carbohydrate Diet score and Osteoporotic fractures risk. Subgroup and interaction analyses demonstrated that age, alcohol consumption, and hypertension had moderating effects on this association.

**Conclusion:**

Higher Low-Carbohydrate Diet scores were associated with a greater risk of Osteoporotic fractures, offering a new perspective on the link between dietary patterns and fracture risk.

## Introduction

Osteoporotic fractures (OF), also known as fragility fractures, are affecting a growing number of individuals globally. The lifetime risk of OF is about 20% for men over 50 years of age and 50% for women over in the same age group (1). Moreover, any new fracture in adults aged 50 years or older increases the risk of subsequent fractures, particularly within the first year following the initial event (2). Currently, the majority of individuals who experience an OF are not adequately assessed or treated for their risk of subsequent fractures (3). With ongoing population growth and aging, the annual number of fractures is projected to rise to 3.2 million by 2040, with associated costs exceeding $95 billion (4).

Diet is a modifiable risk factor, and various studies have demonstrated that dietary patterns may influence the incidence of fractures (5). A meta-analysis showed that adherence to a Mediterranean diet may reduce the risk of hip fractures, although the magnitude of risk reduction is modest (6). Current research on the impact of diet on bone health has concentrated on individual dietary components, particularly calcium and protein. An earlier study has shown that vitamin D3 and calcium can reduce the risk of hip and other non-vertebral fractures in older women (7). Furthermore, a meta-analysis found that high-dose vitamin D supplementation (≥ 800 IU/day) effectively reduced the incidence of hip and non-vertebral fractures in adults over than 65 years (8). An Australian study also found that increased calcium and protein intake through the consumption of dairy products can reduce the risk of falls and fractures in nursing home residents (9).

The low-carbohydrate-diet (LCD) score is a newly proposed macronutrient-based dietary scoring method to explore the relationship between diet and disease, and is considered more appropriate for assessing the risk of chronic diseases (10). The LCD score accounts for the proportional composition of all major dietary macronutrients, dividing fat, protein, and carbohydrates into 11 levels based on their percentage of total energy intake, with each macronutrient assigned a score ranging from 1 (minimum) to 10 (maximum). For carbohydrates, the scoring is reversed, with a minimum score of 10 and a maximum score of 0. The total LCD score is calculated by summing the scores of the three macronutrients (11). Previous studies have demonstrated associations between the LCD score and various health outcomes, including obesity, metabolic syndrome, diabetes, mental disorders, and cognitive performance in the older adults (10–15). However, no studies have yet investigated the relationship between the LCD score and the risk of OF.

The objective of this research is to use data from the NHANES to examine the association between LCD score and the risk of OF, as well as to analyze differences according to gender, age, lifestyle factors, and chronic disease status. The findings may offer new recommendations for dietary intake among middle-aged and older adult populations and provide a reference for effective prevention of OF in these groups.

## Methods

### Data sources and study population

In this cross-sectional study, participant information was sourced from the NHANES database. The NHANES data offers a representative sample of the noninstitutionalized population within the United States.

**Figure 1:**
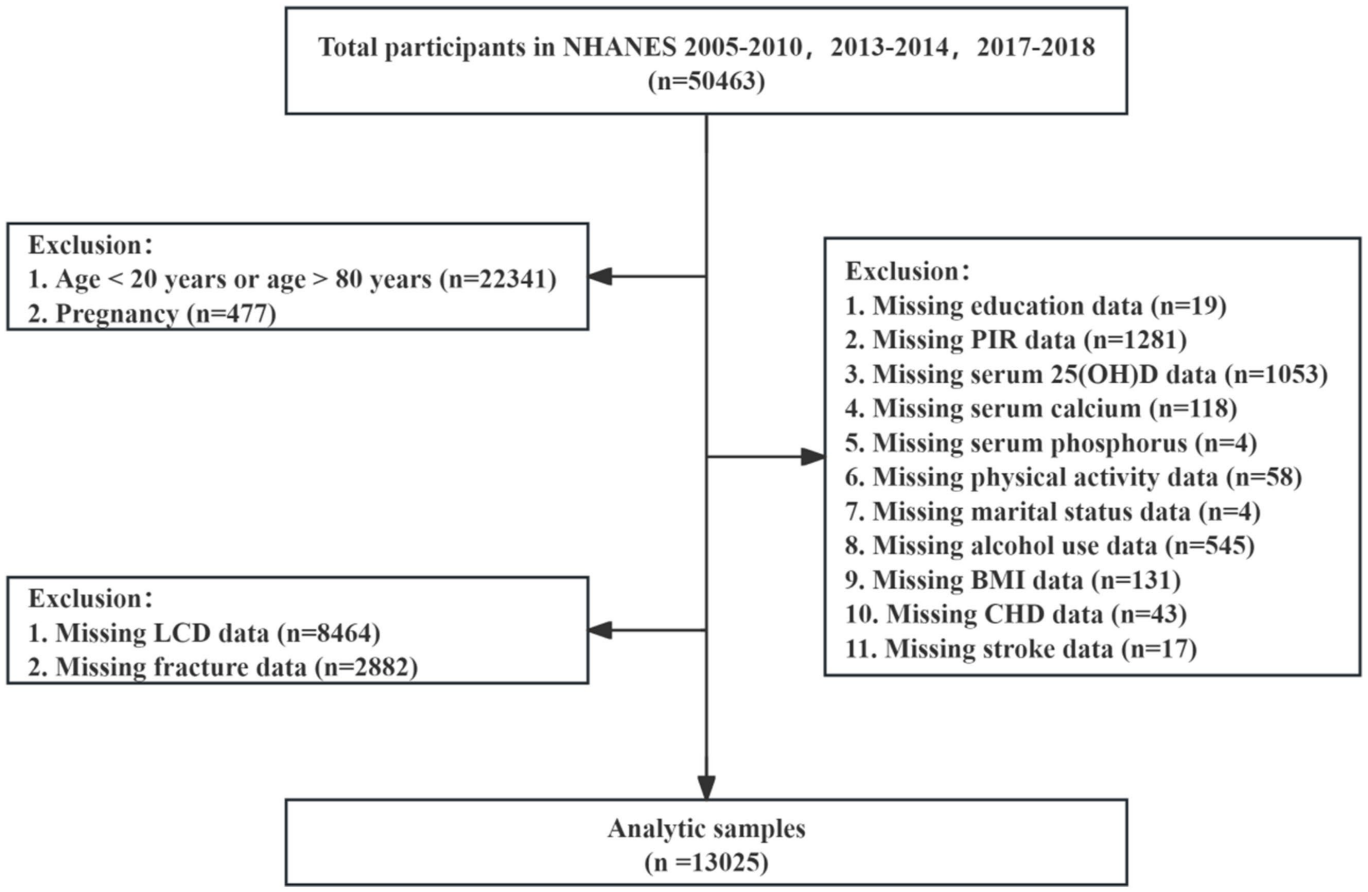
Flow diagram of selection of study participants.

NHANES implements a multi-stage stratified sampling strategy grounded in probability principles, with data collection occurring every 2 years to maximize precision and representativeness. This research analyzed data from NHANES surveys administered in the periods 2005–2010, 2013–2014, and 2017–2018, comprising 50,463 participants. Participants were excluded for the following reasons: (1) age younger than 20 or older than 80 years; (2) pregnancy; (3) missing data on the primary exposure or outcome, specifically LCD score or fracture history; and (4) missing data on covariates (education, poverty income ratio, serum calcium, serum phosphorus, serum 25-hydroxyvitamin D [25(OH)D], physical activity, marital status, alcohol use, body mass index, coronary heart disease, or stroke). After applying these criteria, 13,025 participants remained in the final analytic sample (Figure 1).

### Calculation of the LCD score

The average dietary intake was assessed through two 24-h dietary recall interviews, concentrating on the consumption of fat, protein, carbohydrates, and total energy. Dietary intake data were derived from the NHANES dietary interview component. Trained bilingual interviewers administered 24-h recalls in private rooms at the Mobile Examination Centers (MEC) using standardized measuring guides to estimate portion sizes. Since 2002, a second recall has been conducted 3–10 days later by telephone. Dietary data were collected using the U.S. Department of Agriculture’s Automated Multiple-Pass Method (AMPM), and nutrient intakes were calculated with the USDA Food and Nutrient Database for Dietary Studies (FNDDS). We used the NHANES-provided daily carbohydrate, protein, and fat intakes to compute the LCD score according to the established formula.

The LCD score was established through a thorough evaluation of these three macronutrients. Initially, the quantities of fat, protein, and carbohydrates (measured in grams) were converted into kilocalories using established conversion factors (9 kcal/g for fat and 4 kcal/g for both protein and carbohydrates). Subsequently, the proportion of total energy derived from each macronutrient was calculated. Participants with the highest percentage of energy intake from fat and protein were assigned a score of 10, while those with the lowest received a score of 0. Conversely, for carbohydrates, individuals with the lowest percentage of energy intake were awarded a score of 10, and those with the highest received a score of 0. The final LCD score was the cumulative total of these three nutrient scores, resulting in a possible score range from 0 to 30. A higher LCD score indicated an increased intake of fat and protein, accompanied by a decreased consumption of carbohydrates (11). In this analysis, participants were divided into four categories according to the quartiles of their LCD scores: < 4 points, 4–10 points, 10–16 points, and >16 points.

### OF assessment

Osteoporotic fractures were ascertained through personal interviews. During the NHANES survey, trained interviewers conducted computer-assisted personal interviews (CAPI) in participants’ homes. Self-reported osteoporotic fractures were identified by asking participants: “Has a doctor ever told you that you had a fracture of the hip, wrist, or spine?” Responses indicating “yes” were classified as positive. It is important to note that the reliance on self-reported data may introduce recall bias, which should be considered when interpreting the findings (16).

### Selection of covariates

The study incorporated various covariates that may potentially affect the relationship between the LCD score and the risk of OF. The covariates examined in this research included: age, race/ethnicity, educational attainment, poverty income ratio(PIR), gender, serum 25(OH)D (nmol/L), serum calcium (mg/dL), serum phosphorus (mg/dL), milk product consumption, smoking status, physical activity levels, marital status, alcohol consumption, Body Mass Index (BMI), and the presence of hypertension, diabetes, Coronary Heart Disease (CHD), stroke and total energy intake. For comprehensive definitions of these covariates, please refer to the Supplementary material.

### Statistical analysis

Owing to the survey’s intricate sampling design, all statistical analyses incorporated sampling weights. Continuous variables were summarized using weighted means accompanied by standard deviations, while intergroup comparisons were conducted through weighted t-tests. Categorical variables were represented as weighted percentages and analyzed using weighted chi-square tests. The relationship between LCD scores and OF was examined utilizing weighted logistic regression analyses. Three models were developed: Model 1 was unadjusted; Model 2 included adjustments for gender, age, race, PIR, and education; Model 3 was additionally adjusted for the aforementioned variables as well as smoking status, alcohol consumption, physical activity, marital status, BMI, hypertension, diabetes, serum calcium, serum phosphorus, serum 25(OH)D, milk product consumption, CHD, and stroke, energy. To assess potential effect modification by clinically relevant factors, subgroup analyses were performed across strata of gender, age, BMI, physical activity, smoking and drinking status, hypertension, diabetes, CHD, and stroke. These subgroup variables were pre-specified based on their clinical relevance and previous literature suggesting their potential role in modifying dietary effects on bone health. Additionally, restricted cubic spline (RCS) analyses were applied to evaluate both linear and nonlinear relationships between LCD scores and OF risk. All statistical analyses were performed using R software (version 4.2.1), with *p* < 0.05 considered statistically significant.

## Results

### Baseline characteristics

In total, 13,025 participants (6,353 men and 6,672 women; average age: 53.20 years) were incorporated into the analysis. The clinical characteristics of the participants, categorized based on their OF status, are presented in Table 1.

**Table 1:** The characteristics of participants according to fracture.

Characteristics	Level	Fracture (*n* = 3,024)	No-Fracture (*n* = 10,001)	*p* value
Age (years)		55.47 ± 14.31	49.61 ± 16.34	<0.001
BMI (kg/m^2^)		29.58 ± 6.76	28.90 ± 6.73	0.001
PIR		3.16 ± 1.64	3.13 ± 1.63	0.625
Serum 25(OH)D (nmol/L)		72.58 ± 28.87	69.16 ± 27.53	<0.001
Serum calcium (mg/dL)		9.45 ± 0.39	9.44 ± 0.36	0.477
Serum phosphorus (mg/dL)		3.79 ± 0.56	3.78 ± 0.57	0.693
LCD		11.47 ± 7.58	10.46 ± 7.37	0.001
Total energy(Kcal)		2102.16 ± 21.80	2070.70 ± 12.70	0.1743
Race (%)	Mexican American	4.9	8.1	<0.001
	Other Hispanic	3.1	4.8	
	Non-Hispanic White	80.9	69.8	
	Non-Hispanic Black	7.3	11.2	
	Other Race	3.9	6	
Education (%)	Below high school	13.5	16.2	0.012
	High School or above	86.5	83.8	
Gender (%)	Male	50.5	46.7	0.027
	Female	49.5	53.3	
Milk product consumption (%)	Never	17.6	17	0.979
	Often	38.8	39.3	
	Rarely	14.8	15	
	Sometimes	28.5	28.5	
	Varied	0.3	0.3	
Smoke (%)	Smoker	23	18.7	<0.001
	Smoked	30.7	25.4	
	No-smoke	46.3	56	
Physical activity (%)	High	53.6	57.3	0.022
	Low	46.4	42.7	
Marital (%)	Yes	66.2	65.3	0.537
	No	33.8	34.7	
Alcohol use (%)	Yes	82.8	78.1	<0.001
	No	17.2	21.9	
Hypertension (%)	Yes	48.1	39.5	<0.001
	No	51.9	60.5	
Diabetes (%)	Yes	15.2	13.7	0.173
	No	84.8	86.3	
CHD (%)	Yes	6.8	4.5	<0.001
	No	93.2	95.5	
Stroke (%)	Yes	6.2	3.8	<0.001
	No	93.8	96.2	

LCD, low-carbohydrate-diet score; PIR, Poverty Income Ratio; BMI, body mass index; CHD, coronary heart disease.

In comparison to participants without OF, those with OF were older and exhibited higher LCD scores, elevated serum 25(OH)D concentrations, and greater BMI values (all *p* < 0.05). No substantial differences were found between the two groups regarding PIR, serum calcium, serum phosphorus, dairy product intake, marital status, or prevalence of diabetes (all *p* > 0.05). Nonetheless, statistically significant disparities were noted in race, educational level, gender, smoking and alcohol consumption status, physical activity, hypertension, CHD, and stroke (all *p* < 0.05).

### Associations between LCD score and OF

In order to assess the association between LCD and the risk of OF, we constructed three weighted logistic regression models, as shown in Table 2. The analysis of the LCD score as a continuous variable revealed a consistent and significant relationship with the risk of OF across all three models. Specifically, an increase of one point in the LCD score was linked to a 1.13% elevation in the risk of OF.

**Table 2:** Associations between LCD score and OF.

LCD	Model 1	Model 2	Model 3
	OR (95%CI) *p*-value	OR (95%CI) *p*-value	OR (95%CI) *p*-value
Continuous	1.0182 (1.0083, 1.0281) 0.0005	1.0147 (1.0053, 1.0243) 0.0030	1.0113 (1.0015–1.0212) 0.0240
Interquartile			
Q1 < 4.00	Ref	Ref	Ref
Q2 4.00 ~ 10.00	1.1248(0.9287, 1.3623) 0.2328	1.0669(0.8780, 1.2963) 0.5172	1.0573 (0.8494–1.3161) 0.6111
Q3 10.00 ~ 16.00	1.2280(0.9918, 1.5205) 0.0635	1.1211(0.9142, 1.3749) 0.2762	1.1209 (0.9274–1.3548) 0.2318
Q4 > 16.00	1.4467(1.1990, 1.7454) 0.0002	1.3274(1.1075, 1.5910) 0.0032	1.2248 (1.0212–1.4688) 0.0295
*p* for trend	1.1216(1.0505, 1.1976) 0.0010	1.0872(1.0203, 1.1584) 0.0120	1.0695 (1.0058–1.1372) 0.0326

Model 1: Unadjusted; Model 2: Adjusted for gender, age, race, PIR, education; Model 3: Adjusted for gender, age, race, PIR, education, smoking and drinking status, physical activity, marital status, BMI, hypertension, diabetes, serum calcium, serum phosphorus, serum 25(OH)D, milk product consumption, CHD, stroke and total energy.

**Figure 2:**
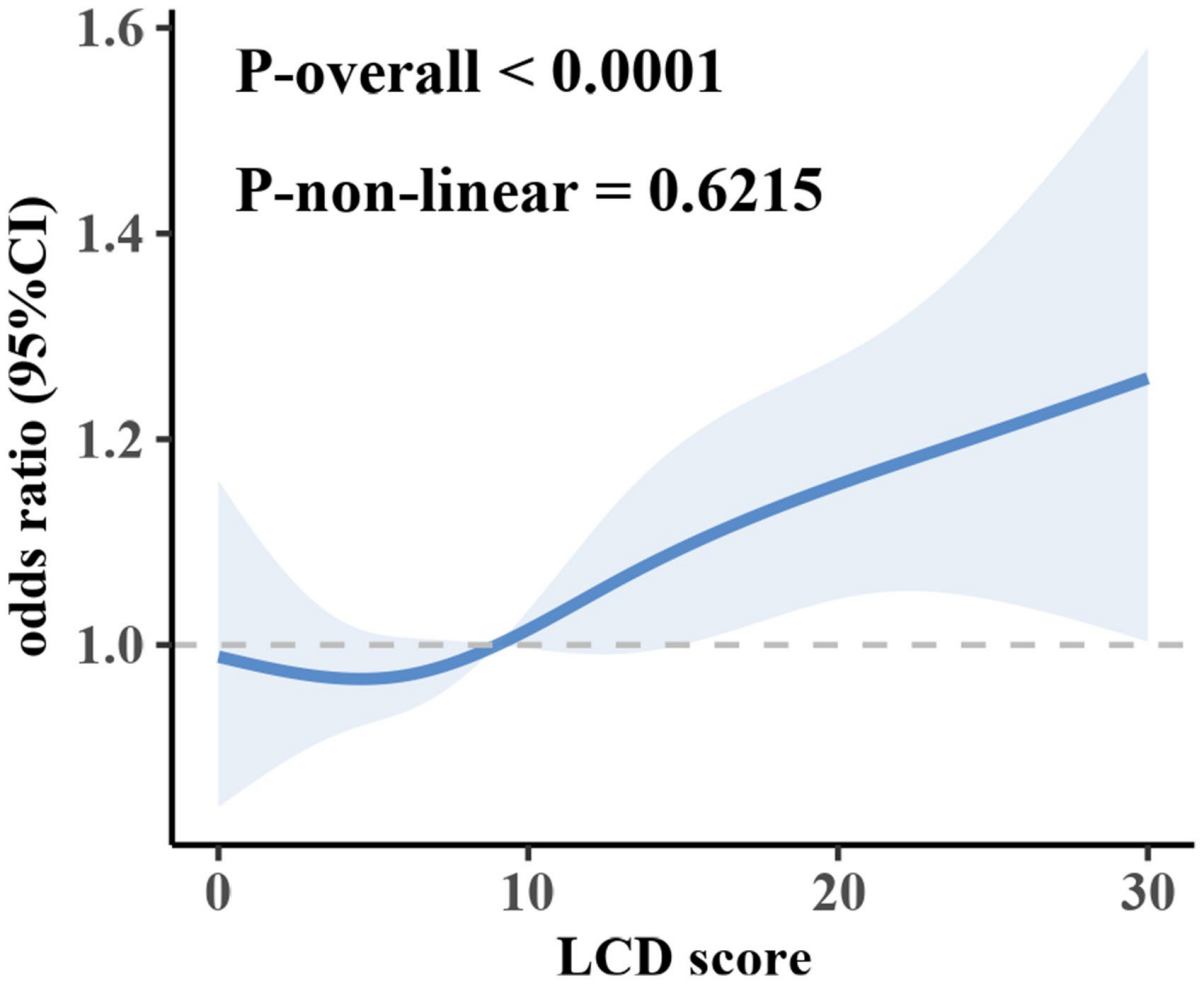
Association of the LCD score and the risk of OF.

Furthermore, the LCD score was stratified into quartiles, with the lowest quartile (Q1) designated as the reference group to evaluate associations with OF risk. In the multivariable-adjusted model, the odds ratios (ORs) for OF in Q2, Q3, and Q4 were 1.0573 (95% CI: 0.8494–1.3161), 1.1209 (95% CI: 0.9274–1.3548), and 1.2248 (95% CI: 1.0212–1.4688), respectively. These findings indicate that higher LCD scores are linked to a greater risk of OF (*p* for trend < 0.05). RCS analysis did not provide evidence of a significant nonlinear relationship between LCD and OF (Figure 2), suggesting a predominantly linear relationship.

### Subgroup analysis

**Figure 3:**
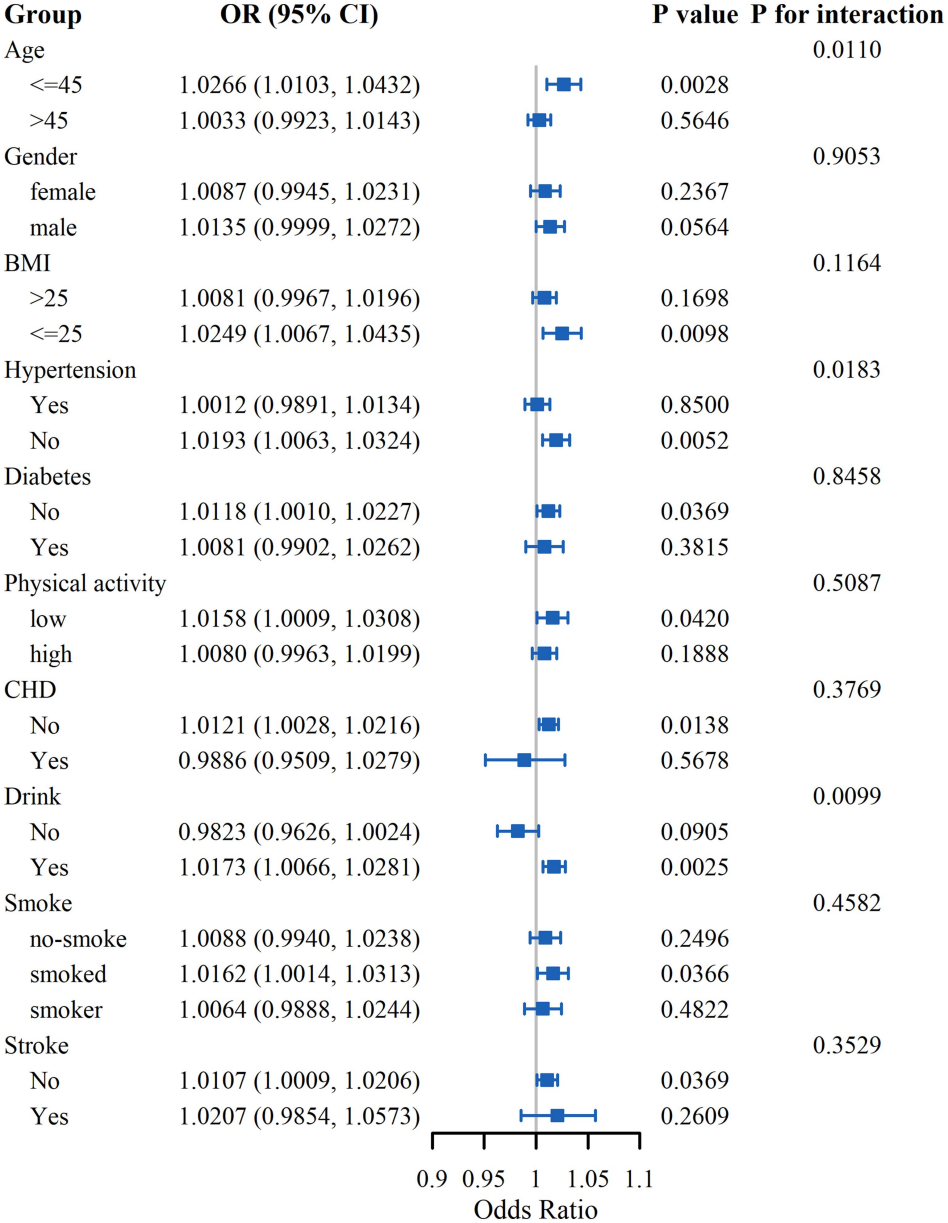
Subgroup analysis of the association between LCD score and OF risk.

In subgroup analyses, we examined the influence of gender, age, BMI, physical activity, smoking and drinking status, hypertension, diabetes, CHD, and stroke on the association between LCD score and OF risk (Figure 3). The results indicated significant interactions of age, drinking status, and hypertension with LCD score in relation to OF risk. In the age ≤45 subgroup, the OR was 1.0266 (95% CI: 1.0103–1.0432), while in the >45 group, the OR was 1.0033 (95% CI: 0.9923–1.0143), with an interaction *p*-value of 0.0110, indicating a significant age-related effect modification. Regarding drinking status, the OR among drinkers was 1.0173 (95% CI: 1.0066–1.0281), compared to 0.9823 (95% CI: 0.9626–1.0024) among non-drinkers, with an interaction *p*-value of 0.0099, suggesting a moderating effect of alcohol consumption. For hypertension, the OR in the hypertensive group was 1.0012 (95% CI: 0.9891–1.0134), and in the non-hypertensive group, it was 1.0193 (95% CI: 1.0063–1.0324), with an interaction *p*-value of 0.0183, indicating that hypertension also moderated the association. The analysis did not reveal any notable interactions within the remaining subgroups.

## Discussion

This study utilized the extensive NHANES dataset to comprehensively examine the relationship between LCD score and the OF risk. The findings revealed that individuals in Q4 of LCD score exhibited a significantly elevated risk of OF in comparison to those in Q1, even after controlling for various covariates. This association was especially evident among participants under the age of 45, those who consumed alcohol, and participants without hypertension. Additionally, RCS analysis suggested a linear correlation between LCD score and the OF risk.

Although the OR for fracture comparing the highest versus lowest quartile of LCD was statistically significant (OR = 1.22), the effect size remains relatively modest. In contrast, well-established risk factors such as age and BMI consistently demonstrate stronger associations with musculoskeletal outcomes. For example, one previous study found that men who transitioned from obesity to a non-obese status over a 10-year period experienced an 81.6% reduction in osteoporosis risk (OR = 0.184, 95% CI: 0.037–0.914, *p* = 0.039) and a 69.8% reduction in wrist fracture risk (OR = 0.302, 95% CI: 0.120–0.757, *p* = 0.012) (17). These comparisons suggest that while LCD may represent a potential risk factor for fracture, its effect is likely secondary to major determinants such as weight history. Thus, the clinical relevance of LCD should be interpreted within the context of these more influential factors.

The findings of this study further suggest that the observed association between LCD score and OF risk may be driven primarily by dietary composition, rather than carbohydrate restriction per se. A meta-analysis reported no significant association between carbohydrate intake and fracture risk when comparing the highest and lowest consumption groups (18). In contrast, recent evidence indicates that higher protein intake may be associated with a reduced risk of hip fracture under certain conditions (19). Fat quality also appears to play an important role:higher intake of saturated fatty acids has been linked to increased fracture risk (20). Whereas moderate linoleic acid intake and elevated circulating levels of polyunsaturated fatty acids—particularly omega-3 fatty acids such as EPA—have been associated with reduced risk (21). Taken together, these findings suggest that the LCD score reflects a complex dietary pattern, and its association with fracture risk likely arises from the combined effects of multiple macronutrients rather than any single nutrient.

Furthermore, previous studies have indicated that LCD can negatively affect high-intensity endurance exercise performance (22, 23). Additionally, compliance with a low-carbohydrate, high-fat dietary regimen has been associated with elevated levels of low-density lipoprotein cholesterol among healthy, normal-weight young women (24). Young individuals typically exhibit more active bone metabolism and higher levels of physical activity, making them more sensitive to nutritional fluctuations. This may explain why LCD score has a more pronounced effect on fracture risk among individuals under 45 years old. Meta-analyses of prospective cohort studies have consistently shown that alcohol consumption is positively associated with overall fracture risk (25, 26), and when combined with a low-carbohydrate diet, may further exacerbate fracture risk. Conversely, in populations with hypertension, certain antihypertensive medications, such as thiazide diuretics, may reduce fracture risk (27, 28), and patients with chronic conditions may pay greater attention to dietary quality to manage their disease. Consequently, the influence of the LCD score on the risk of OF is more pronounced in non-hypertensive populations. However, it is important to note that these subgroup analyses were exploratory in nature, and no correction for multiple testing (e.g., Bonferroni or FDR) was applied. Therefore, the observed interaction effects should be interpreted as hypothesis-generating and require confirmation in future studies.

Additionally, the interdependent nature of nutrients must be considered, as their effects may be influenced by multicollinearity among dietary components, complicating the assessment of individual nutrient impacts. The assessment of bone health and the associated risk of hip fractures is influenced not only by the isolated effects of individual nutrients but also by their interactions, the overall dietary intake, and the individual’s nutritional status (29). The LCD score employed in this study comprehensively evaluates dietary patterns based on the intake of the three major macronutrients. To the best of our understanding, this study represents the inaugural examination of the correlation between LCD score and the risk of OF. Assessing comprehensive dietary patterns, rather than concentrating exclusively on specific nutrients, may provide more actionable guidance for individuals seeking to reduce femur fracture risk through dietary modification. Nevertheless, due to the inherent complexity of dietary patterns, further research is required to clarify how different dietary patterns influence fracture risk.

This research presents several limitations. Firstly, its cross-sectional design limits the ability to infer causality between LCD score and OF risk. Second, both the covariates and the calculation of LCD score were primarily based on self-reported questionnaire data, which may introduce recall or interview bias. Additionally, participants with incomplete data were excluded from the analysis, and it remains unclear whether their exclusion may have influenced the results. Finally, as the data were derived from a U.S. population, the applicability of these findings to other geographical regions or demographic groups necessitates additional exploration.

## Conclusion

This study identified a significant relationship between LCD score and OF risk, with age, alcohol intake, and hypertension status serving as key moderating variables. The observed linear relationship indicates that individuals under 45 years of age, those who consume alcohol, and those without hypertension should pay particular attention to adjusting the proportions of macronutrient intake in their diets. Due to the complexity of dietary nutrition, further research is warranted to elucidate the relationship between dietary patterns and OF risk.

## Data Availability

The data and materials in the current study are available from the corresponding author on reasonable request.
