# Supplementary material for: Association of low-carbohydrate-diet score and osteoporotic fractures: National Health and Nutrition Examination Survey
Source: Front Public Health. 2025 Oct 8;13:1668024. doi: 10.3389/fpubh.2025.1668024 (PMC12540085; doi:10.3389/fpubh.2025.1668024)
Supplement: Supplementary file 1 [file Table_1.DOCX]

**Supplementary Material**

**Association of low-carbohydrate-diet score and** **osteoporotic fractures: National Health and Nutrition Examination Survey**

**Supplementary Methods**

**Variable Definition**

**Smoking status** was divided into three categories: never (smoked less than 100 cigarettes in life), former (smoked more than 100 cigarettes in life and smoke not at all now), now (smoked moth than 100 cigarettes in life and smoke some days or every day).

**Alcohol consumption status** was determined based on responses to the question, “Had at least 12 alcohol drinks in any one year?” Participants who answered “yes” were classified as drinkers, while those who replied “no” were categorized as nondrinkers.

**Vigorous physical activity** was assessed using two items from the physical activity questionnaire: (1) “Does your work involve vigorous-intensity activity that causes large increases in breathing or heart rate, such as carrying or lifting heavy loads, digging, or construction work for at least 10 minutes continuously?” and (2) “Do you do any vigorous-intensity sports, fitness, or recreational activities that cause large increases in breathing or heart rate, such as running or basketball, for at least 10 minutes continuously?” Participants who answered “yes” to either question were classified as engaging in vigorous physical activity.

**Hypertension** was defined as self-reported hypertension, systolic blood pressure ≥140 mmHg, diastolic blood pressure ≥90 mmHg, or current use of antihypertensive medications.

**Diabetes** was defined as self-reported diabetes, use of insulin or hypoglycemic medications, and/or a glycohemoglobin level ≥6.5%.

**Coronary heart disease (CHD)** was identified by a positive response to the question, “Has a doctor or other health professional ever told you that you had CHD?”

**Stroke** was defined as a positive response to the question, “Has a doctor or other health professional ever told you that you had a stroke?”
